# Characterization and fine mapping of a new dwarf mutant in *Brassica napus*

**DOI:** 10.1186/s12870-021-02885-y

**Published:** 2021-02-26

**Authors:** Xin Li, Fujiang Xiang, Wei Zhang, Jindong Yan, Xinmei Li, Ming Zhong, Piao Yang, Caiyan Chen, Xuanming Liu, Donghai Mao, Xiaoying Zhao

**Affiliations:** 1 College of Biology, Hunan Hybrid Rape Engineering and Technology Research Center, Hunan University, Changsha, 410082 China; 2 Shenzhen Institute, Hunan University, Shenzhen, 518057 China; 3 Key Laboratory of Agro-Ecological Processes in Subtropical Region, Institute of Subtropical Agriculture, Chinese Academy of Sciences, Changsha, 410125 China; 4 College of Agronnomy, Hunan Agricultural University, Changsha, 410128 China

**Keywords:** *Brassica napus*, Dwarf, Grain yield, BSA, Fine mapping

## Abstract

**Background:**

Plant height is an important plant characteristic closely related to yield performance of many crops. Reasonable reduction of plant height of crops is beneficial for improving yield and enhancing lodging resistance.

**Results:**

In the present study, we described the *Brassica napus* dwarf mutant *bnd2* that was isolated using ethyl methanesulfonate (EMS) mutagenesis. Compared to wild type (WT), *bnd2* exhibited reduced height and shorter hypocotyl and petiole leaves. By crossing the *bnd2* mutant with the WT strain, we found that the ratio of the mutant to the WT in the F_2_ population was close to 1:3, indicating that *bnd2* is a recessive mutation of a single locus. Following bulked segregant analysis (BSA) by resequencing, *BND2* was found to be located in the 13.77–18.08 Mb interval of chromosome A08, with a length of 4.31 Mb. After fine mapping with single nucleotide polymorphism (SNP) and insertion/deletion (InDel) markers, the gene was narrowed to a 140-Kb interval ranging from 15.62 Mb to 15.76 Mb. According to reference genome annotation, there were 27 genes in the interval, of which *BnaA08g20960D* had an SNP type variation in the intron between the mutant and its parent, which may be the candidate gene corresponding to *BND2*. The hybrid line derived from a cross between the mutant *bnd2* and the commercial cultivar L329 had similar plant height but higher grain yield compared to the commercial cultivar, suggesting that the allele *bnd2* is beneficial for hybrid breeding of lodging resistant and high yield rapeseed.

**Conclusion:**

In this study, we identified a novel dwarf mutant of rapeseed with a new locus, which may be useful for functional analyses of genetic mechanisms of plant architecture and grain yield in rapeseed.

**Supplementary Information:**

The online version contains supplementary material available at 10.1186/s12870-021-02885-y.

## Background

*Brassica napus* (rapeseed) is a major oil crop that is vital for ensuring a supply of edible oil, improving food structure, and promoting the development of aquaculture and light textile industry [1]. *B. napus* belongs to the *Brassica* oil crops of *Cruciferae* [2]. It is a heterotetraploid plant species formed by natural distant hybridization of two basic diploid species of *B. rapa* and *B. oleracea*. The genome of *B. napus* contains approximately 100,000 protein coding genes [3]. The plant exhibits increased height among the conventional rapeseed varieties, and its height is increased by more than 20 cm on average in the hybrid varieties due to widespread heterosis in *B. napus* [4]. A serious problem that framers encounter with tall plants is that they are prone to lodging. As plant height increases, it becomes a major factor restricting yield increase and mechanized harvesting of rapeseed oil [5]. More than 60% reduction in yield was reported to be caused by lodging as a result of increased plant height in *B. napus* [6]. Dwarfism of *B. napus* is crucial for increasing both lodging resistance and yield production [7]. However, dwarf phenotypes are sometimes associated with poor agronomic traits, resulting in poor yield. Therefore, it is of great value for rapeseed breeding to cultivate varieties which can be used for cross breeding with no change in plant height and that perform better in terms of yield.

To date, many dwarf mutants have been identified, but the alleles useful for breeding are rare in rapeseed. For example, the *B. napus* dwarf mutant *NDF-1* was approximately 70 cm tall, and all agronomic characteristics except for seed weight were much less favorable than its original parents. The decrease in the number of siliques per plant as well as the number of seeds per silique led to a decline in yield [8]. Mutant *bndf-1* with a height of 75 cm had more branches, but the plants were too short, resulting in fewer siliques and lower yield [9]. The semi-dwarf mutant *ds-1* was only 69.3 cm high, and showed a lower yield per plant due to the decrease in the number of siliques per plant [10]. The semi-dwarf mutant *dw-1*, approximately 95 cm high, showed a higher number of siliques per plant, but the decreased yield per plant was due to significantly lower numbers of seeds per plant [11]. The mutant ‘*GRC1157*’ was only ~ 90 cm at maturity and showed obvious reduction in main inflorescence length, silique numbers per main inflorescence, and seeds per silique [12]. The semi-dwarf mutant *ds-3* with a height of ~ 70 cm displayed fewer total nodes, shorter internodes and main inflorescences, and the position of the first main branch was lower than that of the wild type [13]. There were also some mutants that although shorter than wild type, their yield was unaffected. The dwarf mutant *DW 871* had an average plant height of 139.1 cm, and compared to the homologous high stem strain, had more effective first branches; however, there was no significant difference in the number of effective siliques, the number and weight of seeds per plant, nor in yield per plant [14]. The EMS-mutagenized *sca* mutant with a plant height of ~ 80 cm was derived as a consequence of a mutation in a single semi-dominant gene, which encodes an Aux/IAA protein (BnaA3.IAA7). The mutant had more siliques per plant, with a similar thousand-seed weight, but each silique had fewer seeds resulting in a similar yield per plant compared to wild type [15]. In addition, the mutants *Bndwf1* [16], *ds-4* [17], and *G7* [18] had heights of 80–110 cm, 23.4 cm, and 30 cm, respectively, but there was no more description of yield-related traits. In this study, we described the dwarf mutant *bnd2* (*B. napus dwarf 2*) generated by EMS mutagenesis [19]. The *bnd2* mutant showed a reduction in plant height, and grain yield compared to wild type. However, the hybrid line F_1_ produced by crossing the mutant *bnd2* with the commercial variety L329, showed no increase in plant height, but showed an increase in grain yield compared to the variety L329, suggesting that *bnd2* was a new locus for plant dwarfism and is useful for hybrid breeding of lodging resistant and high yielding in *B. napus*.

Bulked segregant analysis (BSA) is a rapid method used to detect molecular markers associated with target traits in mapping populations [20]. The combination of BSA and Next Generation Sequencing (BSA-seq) accelerates the cloning of genes responsible for important traits [21]. BSA-seq has been successfully used to map important agronomic traits in many crops such as rice [22, 23], potato [24], and soybean [25]. In this study, the locus *bnd2* for dwarfism was primarily mapped using BSA-seq. The *bnd2* was then fine mapped into a 140-Kb interval where the candidate gene *BnaA08g20960D* was identified. Our findings provide a foundation for cloning of the *BND2* gene, providing a new locus for conferring lodging-resistance and hybrid breeding in *B. napus*.

## Results

### Phenotypic characteristics of the dwarf mutant *bnd2*

**Fig. 1:**
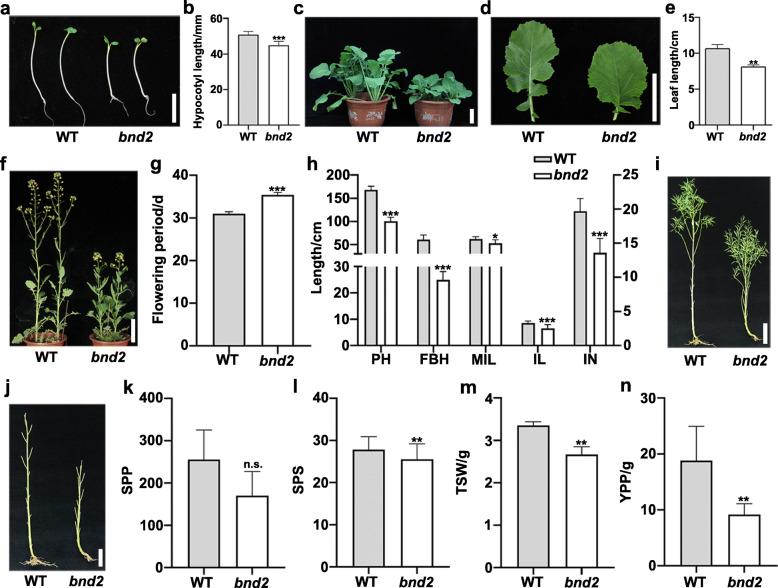
Phenotype characteristics of the mutant *bnd2*. **a-b** The hypocotyls length of one-week-old wild type (WT) and *bnd2* seedlings. **c-e** Plants (**c**) and leaves (**d-e**) of WT and *bnd2* at 5-week-old seedling stage. **f-g** Plants at peak flowering stage (**f**) and flowering period (**g**) of WT and *bnd2*. **h** Effects of the *bnd2* mutant on plant height (PH), first branch height (FBH), main inflorescence length (MIL), internode length (IL) and internode number (IN). **i-j** Comparison of whole plant phenotype (**i**), internodes (**j**) between WT and *bnd2*. **k-n** Comparison of yield-related traits between WT and *bnd2* including siliques per plant (SPP), seeds per silique (SPS), thousand-seed weight (TSW) and yield per plant (YPP). All values in (**b**, **e**, **g**, **h**, **k**-**n**) are mean ± standard deviation (SD) (*n* = 10). Bars = 20 cm in (**a**, **f**, **i**, **j**) and 5 cm in (**c**-**d**). Significance of difference was determined by Student’s *t*-test (n.s., not significant; *, *P* < 0.05; **, *P* < 0.01; ***, *P* < 0.001)

The *B. napus* mutant, *bnd2*, was isolated from EMS-mutagenized seeds of the cultivar “2B” (wild type, WT) [19]. At the seedling stage, *bnd2* showed reduced hypocotyl length and shorter petiole leaves compared to WT (Fig. 1a*-*e, Additional files 1, 2 and 3: Figs. S1–3). At the flowering stage, the *bnd2* mutant exhibited an extremely dwarf and compact stature, and the flowering period of *bnd2* was slightly longer than that of WT (Fig. 1f and g, Additional file 4: Fig. S4). At maturity, the plant height of *bnd2* was 100.65 ± 8.09 cm (*n* = 10), which was 59.8% of WT height (168.2 ± 7.61 cm, *n* = 10) (Fig. 1h and i, Additional file 5: Fig. S5, Additional file 6: Table S1). Furthermore, the first branch height, internode length, internode number, and main inflorescence length of *bnd2* were 41, 76.7, 69, and 85.2% of that of WT, respectively. These results suggested that the dwarf traits were associated with lower position of the first branch, shorter internode length, lower internode number, and reduced main inflorescence length (Fig. 1h-j, Additional files 5 and 7: Figs. S5 and 6, Additional file 6: Table S1). Accordingly, *bnd2* produced lower yield per plant (YPP) (48.4% of WT) due to shorter silique length (83.1% of WT), fewer seeds per silique (SPS) (92.1% of WT), and less thousand-seed weight (TSW) (90% of WT) compared to WT, although similar siliques per plant (SPP) were observed in both *bnd2* and WT (Fig. 1k-n, Additional file 6: Table S1, Additional file 8: Fig. S7).

### Cell elongation and expansion is decreased in stems of *bnd2*

**Fig. 2:**
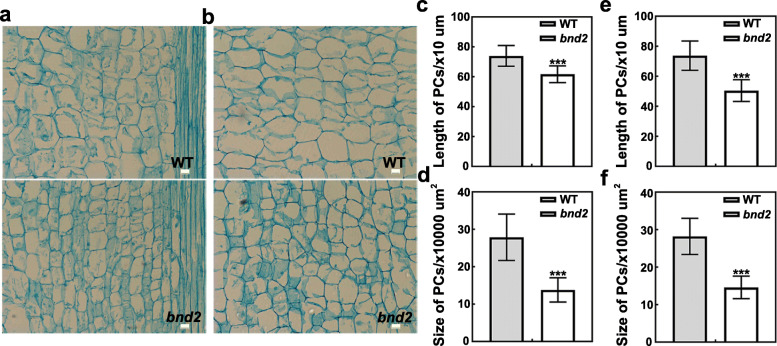
Cell elongation and expansion in stem of the mutant *bnd2*. a-b The longitudinal sections (**a**) and the cross sections (**b**) of parenchyma cells in the 2nd to 3rd internodes of WT and *bnd2.* **c-d** Statistical analysis of the length (**c**) and size (**d**) of the parenchyma cells shown in (**a**). **e-f** Statistical analysis of the length (**e**) and size (**f**) of the parenchyma cells shown in (**b**). All values in c-f are shown as mean ± SD (*n* = 58, 110 of WT and *bnd2* cells in c and d; *n* = 68, 107 of WT and *bnd2* cells in **e** and **f**). Bars = 10 μm. The significance of the difference was determined by Student’s *t*-test (***, *P* < 0.001)

To investigate the underlying cellular basis for the dwarf phenotype in *bnd2*, we performed paraffin sectioning and observed cross and longitudinal sections of the stems of *bnd2* and WT at the early bolting stage. As shown in Fig. 2, the parenchymal cells of *bnd2* were arranged closely and displayed irregular shapes and different sizes compared to WT (Fig. 2a and b). The cell area and cell length were significantly reduced in both cross and longitudinal sections in the *bnd2* plant (Fig. 2c-f). Indeed, in cross and longitudinal sections, the cell area was decreased by 48.2 and 50.5%, and cell length was decreased by 31.6 and 16.6%, respectively. These results suggested that the reduction of parenchyma cell area and length in the plant stem was likely to be the main cause of the dwarfism of the mutant *bnd2*.

### Inheritance of the dwarf phenotype in the mutant *bnd2*

**Fig. 3:**
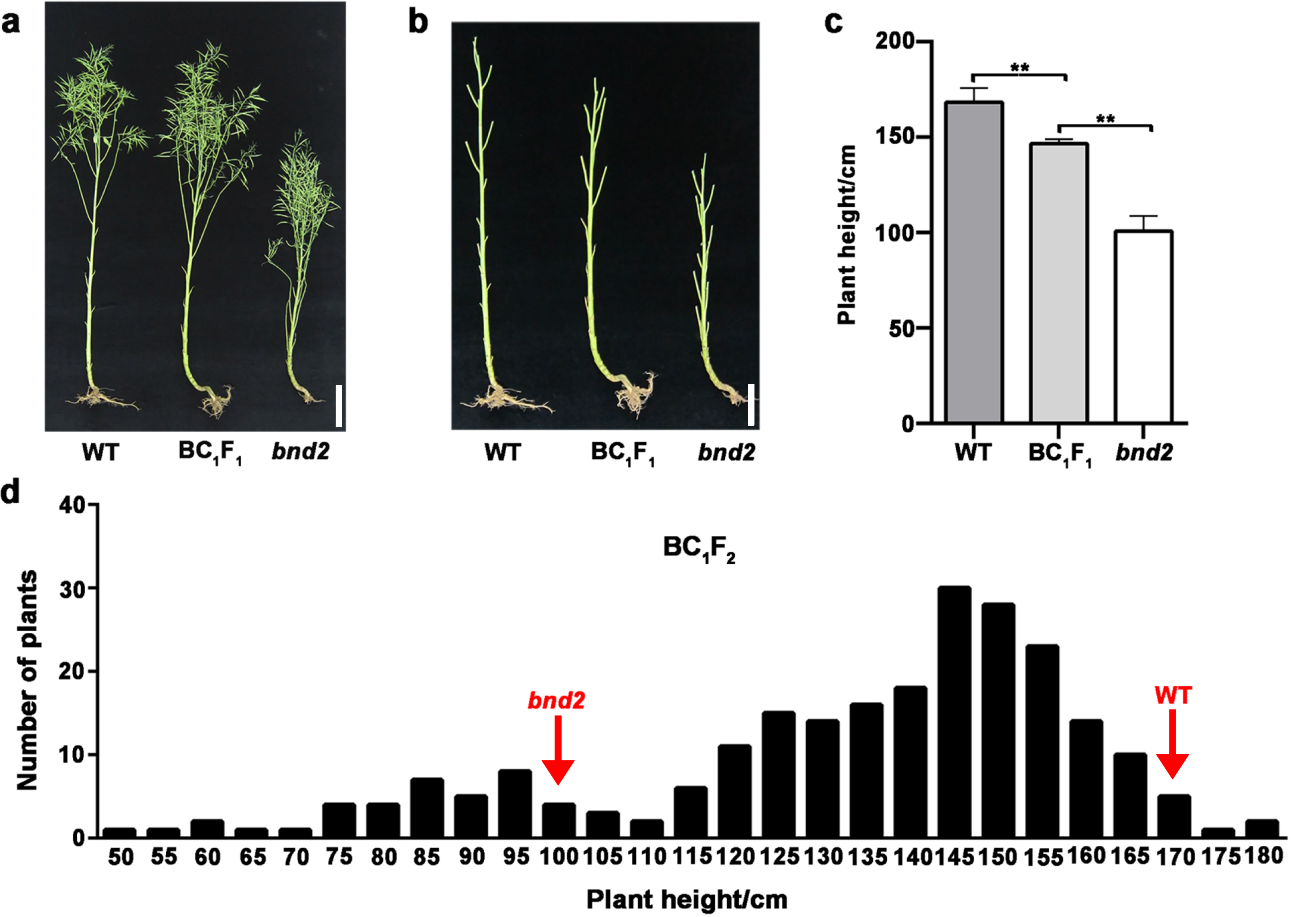
Phenotype and trait inheritance of *bnd2* in the backcross population. **a-b** Performance of WT (left), *bnd2* (right) and their F_1_ hybrid (middle) at maturity. **c** Plant height comparison among WT, *bnd2* and their F_1_ at the maturity stage. Values are shown as mean ± SD (*n* = 25). Bars = 20 cm. The significance of difference was determined by Student’s *t*-test (**, *P* < 0.01). **d** Frequency distribution of plant height in the BC_1_F_2_ population containing 236 individuals derived from the cross of WT and *bnd2*

To analyze the inheritance of the dwarf mutant, *bnd2* was crossed with its original WT parent, and with the commercial cultivar L329. The resulting heterozygous BC_1_F_1_ plants (*bnd2*/WT) displayed an intermediate plant height between that of WT and the mid-parent value, suggesting that the allele *BND2* is semi-dominant to the allele *bnd2* (Fig. 3a*-*c, Additional files 9 and 10: Figs. S8 and 9). In addition, based on the plant height of the BC_1_F_2_ generation of (WT × *bnd2*), the 236 BC_1_F_2_ plants were classified into two groups: the dwarf phenotype of *bnd2* (dwarf plants, *n* = 49), and the tall plant group with a height similar or close to WT (tall plants, *n* = 187). The BC_1_F_2_ generation was in line with an expected Mendelian inheritance ratio of 1:3 (dwarf plants: tall plants, *χ*^2^ = 2.04 < *χ*^2^_0.05,1_ = 3.84) (Fig. 3d). Another F_2_ population was generated from a cross between *bnd2* and the commercial cultivar L329 which possessed a normal plant height of ~ 159 cm. There were 75 plants with a dwarf phenotype and 188 plants with plant height similar or close to that of L329 in the F_2_ population, also showing a Mendelian segregation ratio of 3:1 (tall plants: dwarf plants, *χ*^2^ = 1.46 < *χ*^2^_0.05,1_ = 3.84) (Additional file 11: Fig. S10). Taken together, these results suggested that the dwarf phenotype of *bnd2* was controlled by a single recessive gene.

### Genetic mapping of the dwarf mutant *bnd2* by BSA-seq

**Fig. 4:**
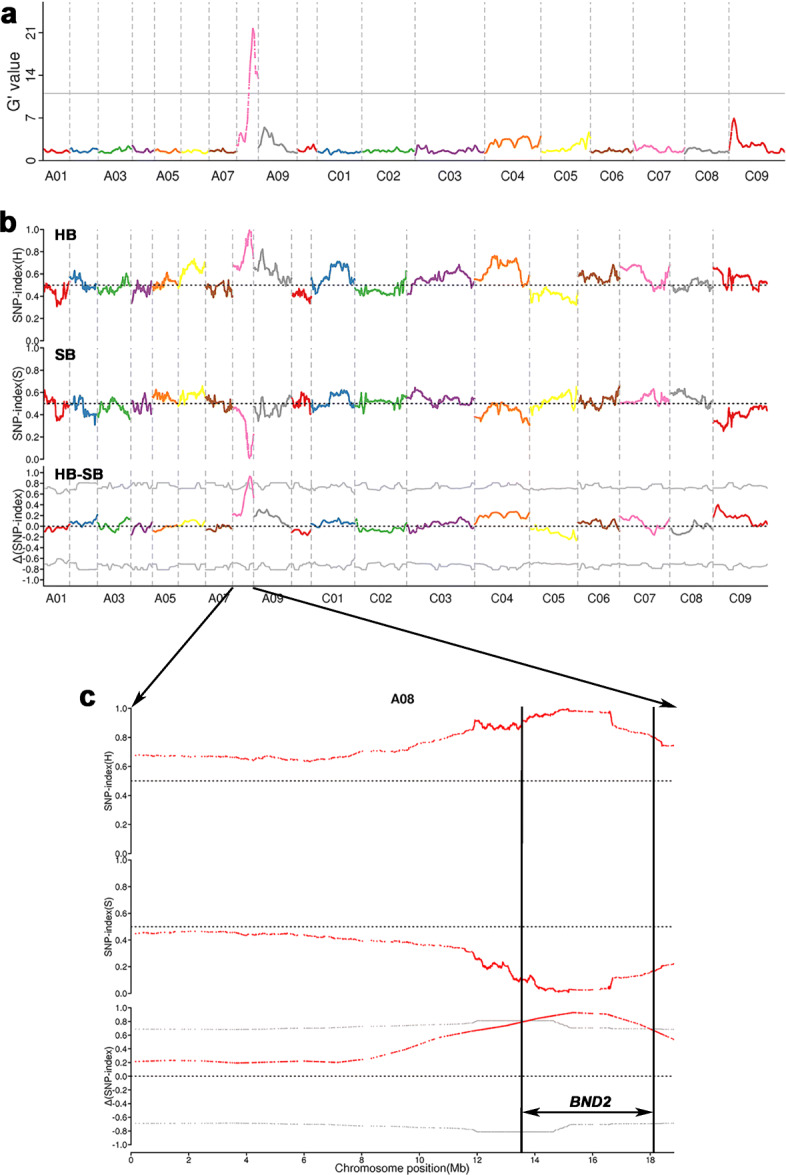
Primary mapping of *BND2* by bulked segregant analysis using resequencing. The physical position (unit: MB) of each chromosome in *Brassica napus* is represented as the x-axis. **a** The G’-value was represented as the y-axis. **b** The SNP index of 3-Mb interval with 10-kb sliding window each time was represented by the y axis. The △(SNP-index) was calculated by subtracting the SNP index of the short bulk (SB) from that of the high bulk (HB). The dotted line was the threshold of △(SNP-index) set as the mean of △(SNP-index) ± 3*SD. **c** The *BND2*-containing genomic interval was identified by using the threshold line

To map the gene conferring the *bnd2* phenotype, the F_2:3_ population derived from a cross between *bnd2* and L329 was used to perform BSA resequencing. In the F_2:3_ population (*n* = 157), 25 extremely dwarf and 23 extremely tall homozygous lines were selected to make a short and high bulk. After sequencing the two bulks and their parents, the total data after quality control filtering was 118.75 Gb, of which 31.21, 26.11, 29.09, and 32.34 Gb corresponded to the L329 parent, the mutant *bnd2* parent, the high bulk, and the short bulk, respectively, with a coverage depth of 24.33 X, 18.55 X, 21.41 X, and 22.35 X, separately (Additional file 12: Table S2). Clean reads of 105,361,953, 89,416,611, 99,097,181 and 109,214,266 were harvested for the L329 parent, the mutant *bnd2* parent, the high bulk, and the short bulk, respectively (Additional file 12: Table S2). The sequencing data showed that the percentage of bases with a quality score of more than 30 (Q30) in two pools and two parents reached more than 92.99%, and Q20 reached more than 97.78% (Additional file 12: Table S2). In addition, the average GC content was 37.35%, and the average genome coverage was 74.57% (Additional file 12: Table S2). Therefore, we consider that the quality of the sequencing data is consistent with expectations and can be used for further analysis. According to alignment with the ‘Darmor-*bzh*’ reference genome [26], 1,157,351 polymorphisms (containing 948,896 single nucleotide polymorphisms (SNPs) and 208,455 insertions/deletions (InDels)) were identified in the two pools. The G’ value and SNP-index were calculated from the short bulk and the high bulk; the ∆(SNP-index) was drawn based on the physical positions of the reference genome (Fig. 4a and b). Only one significant ∆(SNP-index) peak was identified and located in the 4.31 Mb region from 13.77 Mb to 18.08 Mb on chromosome A08 (Fig. 4c), suggesting that it was the candidate locus harboring the *BND2* gene.

### Fine mapping and candidate gene analysis

**Fig. 5:**
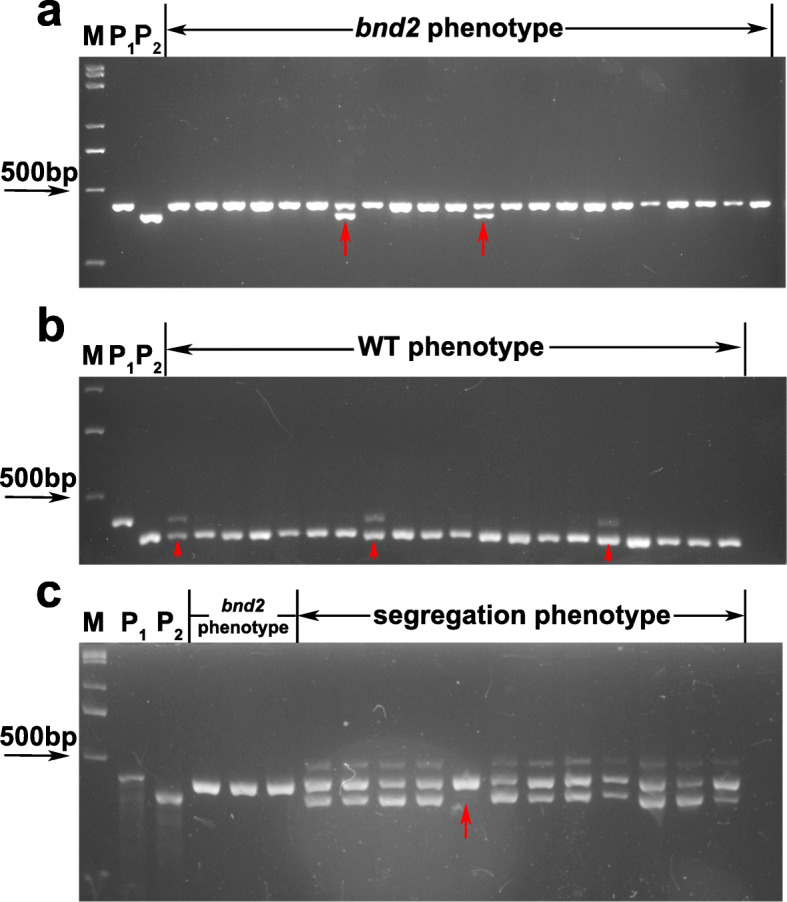
The genotypes of some F_2:3_ lines derived from cross between *bnd2* and L329 at the marker ID1656. **a** The genotypes of the lines with the *bnd2* phenotype. **b** The genotypes of the lines with WT phenotype. **c** The genotype of the lines with phenotype segregation. M means DNA Marker. P_1_ means the mutant parent *bnd2*. P_2_ means the WT parent L329. The red arrow indicates the recombinants between *BND2* and the marker ID1656

**Fig. 6:**
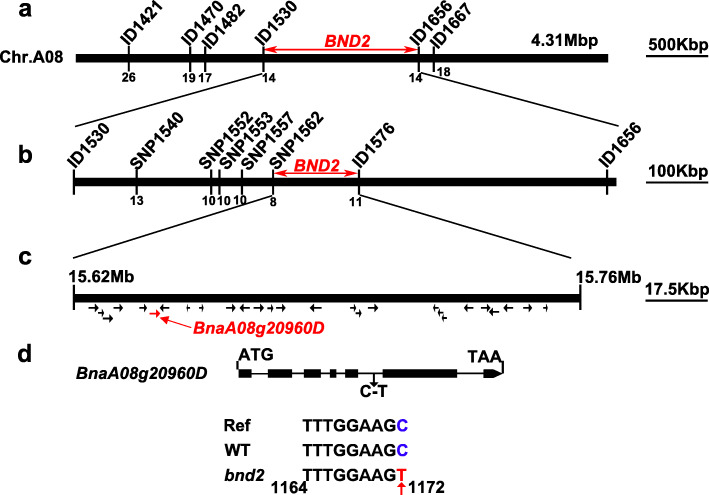
Fine mapping of *BND2*. **a** The *BND2* locus was primary mapped into the interval flanked by the markers ID1530 and ID1656. Numbers below each marker is the number of recombinants. **b** The *BND2* locus was finally mapped to a 140-Kb flanked by SNP1562 and ID1576. **c** Relative physical position of the *BND2* locus. Numbers above chromosome A08 indicate physical distance (unit: Mb). The region contains 27 annotated genes according to the ‘Darmor-*bzh*’ reference genome. The candidate gene, *BnaA08g20960D* is marked in red. **d** Structure of the *BnaA08g20960D* gene, a single nucleotide substitution (C-T) between *bnd2* and its wild type parent 2B was identified in the fifth intron. Exons and introns were represented as black boxes or black lines, respectively

To fine map the *BND2* locus, six InDel markers (ID1421, ID1470, ID1482, ID1530, ID1656, and ID1667) were developed from the 4.31-Mb region harboring *bnd2* based on the BSA-seq result. Then, in the F_2:3_ population (*bnd2*/L329) with 543 lines, the six markers were used to genotype 107 recessive dwarf lines, as well as two controls, including 25 WT lines and 25 heterozygous lines with segregation in plant height (Fig. 5, Additional file 13: Fig. S11). According to fine genotypes of these lines, *bnd2* was further fine mapped into the 1.26-Mb interval flanked by two InDel markers, ID1530 and ID1656 (Fig. 6a). To further narrow the candidate interval, six pairs of new polymorphic markers were developed in the region of *bnd2*, including the SNP markers SNP1540, SNP1552, SNP1553, SNP1557, and SNP1562, and the InDel marker ID1576 (Fig. 6b). Subsequently, *BND2* was narrowed down to an interval from 15.62 Mb to 15.76 Mb, and the physical distance was 140.0 Kb (Fig. 6b). After fine mapping and annotating the information of the reference genome ‘Darmor-*bzh*’, there were 27 genes in the 140 Kb candidate interval, 14 of which were not cloned or had unknown functions (Fig. 6c). By analyzing the annotation results of all mutations in the candidate interval, one SNP occurred in the candidate gene, *BnaA08g20960D* (Fig. 6d), which encodes an inositol-pentakisphosphate 2-kinase family protein, where a single nucleotide change from C to T occurred in the fifth intron region. Moreover, quantitative real-time PCR (qRT-PCR) results showed that the expression of *BnaA08g20960D* in *bnd2* was significantly lower than that of WT (Additional file 14: Fig. S12). Semi-quantitative PCR (semi-qPCR) analysis further confirmed that *BnaA08g20960D* exhibited reduced mRNA expression, although no splicing changes were found in *bnd2* (Additional file 15: Fig. S13). Therefore, we considered this gene as a key candidate gene.

### The potential application of *bnd2* in hybrid rapeseed breeding

**Fig. 7:**
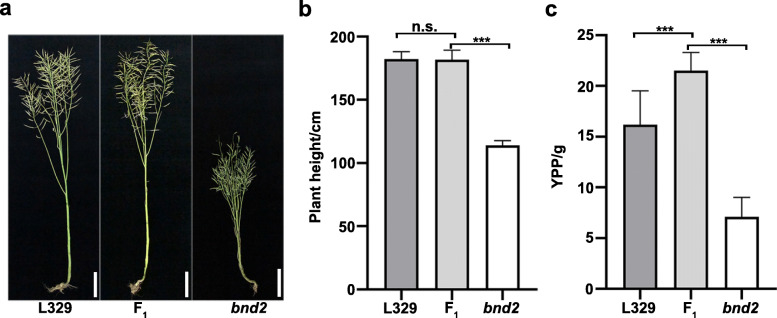
Performance on plant height and grain yield of *bnd2* in the hybrid line. **a** Phenotypes of L329 (left), *bnd2* (right) and their hybrid (F_1,_ middle) at the maturation stage. **b-c** Plant height **(b)** and yield per plant **(c)** of L329, *bnd2* and their F_1_ hybrid at maturity. Values are shown as mean ± SD (*n* = 10). Bars = 20 cm. The significance of difference was determined by Student’s *t*-test (n.s. not significant; ***, *P* < 0.001)

Due to the low yield of *bnd2*, it cannot be used for inbreeding of rapeseed. To test its potential application in hybrid breeding, we crossed the *bnd2* mutant (*bnd2/bnd2)* with the commercial cultivar L329 (*BND2/BND2*) to derive the hybrid line F_1_ (*BND2/bnd2*). The plant height of the F_1_ hybrid was similar to L329 (Fig. 7a and b, Additional file 16: Fig. S14, Additional file 17: Table S3). However, the yield per plant (YPP) of F_1_ was significantly higher than both of *bnd2* and L329, showing an increase of 32.7% compared to L329 due to more seeds per silique (SPS), and three times as much as *bnd2* (Fig. 7c, Additional file 17: Table S3). These results suggested that although the presence of *bnd2* in the hybrid line dose not increase plant height, it induces higher grain yield due to the semi-dominant effect of *BND2* over *bnd2* and the heterosis between the two lines.

## Discussion

Plant height is an important plant characteristic closely related to yield performance of many crops; however, very tall plants tend to have increased risk of lodging. Although many dwarf mutants in rapeseed have been identified and reported, only a few varieties could be used as practical breeding resources [8–18]. Compared with rice and wheat [27], rapeseed dwarf mutants are rare. In this study, we described a new dwarf mutant *bnd2* isolated from EMS-mutagenized seeds of *B. napus* [19]. The mutant *bnd2* displayed a height of approximately 100 cm at maturity. The decrease in plant height was due to lower position of the first branch, shorter internodes, and reduced main inflorescence length (Fig. 1h-j, Additional files 5 and 7: Figs. S5 and 6, Additional file 6: Table S1). The reduced first branch height and main inflorescence length are conducive to lodging resistance [28]. The mutant *bnd2* displayed a poor biological yield performance due to the limitation of its height (Fig. 1k-n), which limits its benefit for inbreeding of high-yield cultivars. While it was reported that a *B. rapa* dwarf mutant *Brrga1-d* showed significant reduction in seed yield, it had no significant influence on the seed yield of hybrid lines containing the dwarf allele in *B. napus* [29]. The relatively short *sca* mutant displayed intermediate height between corresponding parents, and significantly higher YPP after crossing it with the rapeseed cultivars 4312, ZS11, and ZY821 [15]. In this study, as shown in Fig. 3, the heterozygous BC_1_F_1_ plants (*bnd2*/WT) derived from a backcross of *bnd2* with its WT parent, displayed intermediate plant height between that of WT and the mid-parent value (Fig. 3a-c, Additional files 9 and 10: Figs. S8 and 9), suggesting that the allele *BND2* is semi-dominant over the allele *bnd2*. While the F_1_ plants (*bnd2*/L329) derived from crossing *bnd2* with the commercial cultivar L329 showed no significant difference with regards to plant height (Fig. 7a and b, Additional file 16: Fig. S14, Additional file 17: Table S3), the plants displayed a significant increase in grain yield compared to L329 (Fig. 7c, Additional file 17: Table S3), suggesting that by combining the semi-dominant effect of *bnd2* and the heterosis between two lines, the allele *bnd2* may be a potential genetic resource for lodging-resistance and high-yield breeding in hybrid rapeseed.

In the fine mapping interval of *bnd2*, a candidate gene *BnIPK1*, *BnaA08g20960D* (Fig. 6c) was annotated to encode an inositol 1,3,4,5,6-pentapentaphosphate 2 kinase, which catalyzes the terminal step in the biosynthetic pathway of phytic acid (*myo*-inositol-1,2,3,4,5,6-hexakisphosphate [InsP_6_]) [30]. Over the last two decades, with the discovery of *IPK1* in budding yeast [31], *IPK1* homologous genes were subsequently isolated from *Schizosaccharomyces pombe* [32], human [33], *Drosophila* [34], maize [30, 35], and *Arabidopsis thaliana* [36]. As a product of *IPK1*, phytic acid acts not only as a storage compound in seeds, but is also involved in hormones and signal transduction processes [37]. It has been reported that InsP_6_ is a specific functional co-factor of the auxin receptor TIR1 [38] and acts as a ‘conformational stabilizer’ for TIR1 protein [39]. InsP_6_ binds TIR1 and stabilizes the active amino acid residues around auxin, thus ensuring effective signal transduction between TIR1 and auxin [40]. In addition, *AtIPK1* has been reported to be essential for sustaining plant growth. In *Arabidopsis*, the mutant *atipk1–1* displayed reduced size and leaf epinasty [35], while the two mutants *atipk1–2* and *atipk1–3* showed more serious growth retardation [41]. Similarly, Lee et al. found that the *atipk1–1* mutant was significantly smaller than the wild type (Columbia-0) [37]. The seed yield of the *atipk1* mutant was only 52% of that of WT because many pods of the mutant contained abortive seeds [37]. In the present study, *bnd2* showed shorter petiole leaves, decreased and shorter internodes, and decreased plant height and seed yield compared to WT (Fig. 1c-e, h-n, Additional files 2, 3, 5, and 7: Figs. S2, 3, 5, and 6, Additional file 6: Table S1), suggesting that its dwarfism may be related to auxin signal transduction, which needs to be further explored.

It has been reported that introns of many genes have a positive regulatory effect on gene expression [42]. The promoter of the rice gene *OsBP-73* requires the participation of the gene intron sequence to drive the expression of the *GUS* gene in transgenic rice, but the complete *OsBP-73* intron itself has no promoter activity [42]. Liu et al. [43] found that the intron of *BnFAD2-C5* can enhance the transcription level of the promoter. The 5′ UTR of *Arabidopsis AtVTC1* contains an intron sequence, which can promote the expression of *AtVTC1* [44]. In addition, the existence of introns provides a variety of splicing methods for genes. The same DNA sequence can produce different protein products after mRNA transcription by alternative splicing [45]. Sun et al. [30] found that, among 18 maize full-length *ZmIPK1A* cDNA clones in leaves and seeds, 50% of the transcripts had interrupted reading frames due to alternative splicing of introns 6 and 7. However, intron mutations can lead to abnormal splicing, which in turn could result in exon skipping, new exon generation or intron retention [46]. Yuan et al. [47] found that a single G-A point mutation of the soybean *GmIPK1* in the 5′ terminal of intron 5 resulted in the exclusion of the fifth exon, disrupting *GmIPK1* functionality. In this study, we identified a single C-T mutation in the fifth intron of *BnaA08g20960D* in the dwarf mutant *bnd2* (Fig. 6d), and found that the expression of *BnaA08g20960D* was attenuated in *bnd2* (Additional file 14: Fig. S12), although no splicing change was observed between *bnd2* and WT (Additional file 15: Fig. S13). We therefore considered it as the candidate gene for the dwarf phenotype of *bnd2*; however, the molecular basis needs to be further examined.

## Conclusion

In this study, we described a new dwarf mutant *bnd2* isolated using EMS mutagenesis. The mutation of *BND2* decreased plant height and grain yield in the background of the inbred line, but maintained the plant height and increased grain yield in the background of the hybrid line. Through BSA-seq and fine mapping, *bnd2* was mapped to a 140.0-Kb region on chromosome A08 in *B. napus*. In summary, we identified a dwarf mutant *bnd2* which may be useful for hybrid breeding with lodging resistance and high yield, and the fine mapping results will benefit functional analyses of genetic mechanisms of plant architecture and grain yield in rapeseed.

## Methods

### Plant materials and growth

*B. napus* 2B was used as a wild type in this study. 2B is a maintainer line of bolima cytoplasmic male sterile line. The *B. napus* dwarf mutant *bnd2* was isolated and screened from 2B seeds induced by 0.8% EMS solution in our previous study [19]. Another commercial cultivar L329 (Xiangyou 15) described previously [48] was used to construct the F_2:3_ population and the F_1_ hybrid line for *BND2*’s genetic analysis and evaluation of its potential value in hybrid breeding. Plants of all generations including their parents were grown in the filed in Ningxiang, Hunan province.

### Agronomic traits analysis

Plants of all generations including their parents were grown in an irrigated field. Each plot in the field is about 2 m wide, 2 m long, with a row spacing of 33 cm. Ten plants were planted in each row. The agronomic traits were measured and counted at maturity stage. Ten plants from plot were randomly selected for agronomic traits analysis. The plant height (PH), internode length (IL), internode number (IN), first branch height (FBH), main inflorescence length (MIL), number of effective primary branches (NPB), number of siliques on raceme (NSR), siliques per plant (SPP), length of siliques (LS), seeds per silique (SPS), thousand-seed weight (TSW) and yield per plant (YPP), were measured and counted as previously described [49, 50]. Significant differences were determined by Student’s *t*-test using SPSS version 25 (SPSS Inc., Chicago). The segregation ratio was calculated by Chi square test.

### Microscopy analysis

The second internode stem segment from the top to the bottom of *bnd2* and WT plants at the early stage of bolting were fixed in FAA (formalin-acetic acid-alcohol) solution for 16–20 h, and then subjected to dehydration and transparency. The tissues were then immersed and embedded in paraffin wax (Sigma, USA), and sectioned to 6–10 μm (Leica rm2265). After staining with 0.05% toluidine blue, the samples were examined and photographed by a reverse fluorescence phase contrast microscope (Nikon). The stem cell size and number were calculated by the Image J software (http://rsb.info.nih.gov/ij/).

### Genetic mapping and BSA-seq

To map the *BND2* locus, a F_2:3_ mapping population containing 157 lines was obtained from the self-pollinated F_2_ lines, which was derived by the cross between *bnd2* and L329. Young leaves were collected from each 157 F_2:3_ line for genomic DNA extraction using the method of SDS extraction as described by Dellaporta et al [51]. The DNA concentration and purity were detected by Nanodrop one (Thermo Fisher, USA). The DNA of 25 extremely dwarf and 23 extremely tall individuals were mixed to make a short bulk and high bulk, separately.

Both two parents, together with above two bulks, were sequenced by next generation sequencing strategy. The paired end (PE) library was constructed according to the manufacture’s instructions (NEBNext®Ultra™IIFS DNA Library Prep Kit for Illumina®), in which the genomic DNA was randomly broken into 300-500 bp fragments. High-throughput sequencing was performed on Illumina NovaSeq platform to generate average 30 Gb sequence data per sample. Burrows-Wheeler Alignment tool (BWA, version 0.7.15) was used to align the PE reads to the reference genome of ‘Darmor-*bzh*’ v4.1 [26]. And, SAM format was then converted to the BAM format using SAMtools (version 1.3.1). Picard tool (version 1.91) was used to sort the reads in the BAM file and remove polymerase chain reaction (PCR) duplication. Variants including single nucleotide polymorphism (SNP) and insertion/deletion (InDel) were detected by the HaplotypeCaller of Genome analysis toolkit (GATK, version 3.7). The candidate region was determined based on Δ (SNP-index) and G’ value [52] calculated by QTLseqr (version 0.7.5.2) [53], and ANNOVAR (version 2016FeB1) was used to predict the effect of variants on gene function (Wuhan Genoseq Technology Co. Ltd., Hubei, China).

### Development of molecular markers and their genotyping

According to the BSA-seq results and the positions of SNP and InDel on chromosomes contained in the target gene candidate region, and based on the ‘Darmor-*bzh*’ sequence of the *B. napus* reference genome, DNA sequences of SNP/InDel were extracted by extending 250 bp forward (5’ end) and back (3’ end) respectively, and Primer Premier (version 5.0) was used to design SNP/InDel markers. For all markers, two parents L329 and *bnd2* were used for polymorphism screening, and markers with polymorphism were used for PCR amplification and genotype identification of F_2:3_ population. For InDel markers, 3% agarose gel electrophoresis was used to separate PCR products. While for SNP markers, PCR products were first identified by 1% agarose gel electrophoresis and if bands between the *bnd2* and L329 were clear, then sent the PCR products to sequencing (TsingKe Biological Technology Co. Ltd., Changsha, Hunan, China). PCR sequencing results were analyzed with Sequencher (version 5.0). The band type consistent with *bnd2* (P_1_) was recorded as A, the band type consistent with L329 (P_2_) was recorded as B, and both band types were recorded as H, and the deletion was not recorded. The corresponding mapping markers sequences are listed in Additional file 18: Table S4.

### RNA extraction and quantitative real-time PCR (qRT-PCR)

To detect mRNA expression of the *bnd2* candidate gene, *BnaA08g20960D*, seven-day-old seedlings of *bnd2* and wild type 2B grown in soil were sampled. Total RNA was extracted by AG RNAex Pro Reagent (Accurate Biology, China). The cDNA was then synthesized using the HiScript II 1st Strand cDNA Synthesis Kit (+gDNA wiper) (Vazyme, China). qRT-PCR was performed in an ABI StepOne Plus system (Thermo Fisher, USA) using ChamQ universal SYBR qPCR Master Mix (Vazyme, China) according to the manufacture’s instructions. All primers are listed in (Additional file 19: Table S5) and *BnActin7* was used as an internal reference. Each experiment was biologically repeated three times.

### Semi-quantitative PCR (semi-qPCR) analysis

To detect the splicing of the *bnd2* candidate gene, *BnaA08g20960D*, seven-day-old seedlings of *bnd2* and wild type 2B grown in soil were sampled for RNA extraction. 2xTaq Master Mix (Novoprotein, China) was used in PCR reaction according to the manufacture’s instructions. PCR was performed with a 5 min denaturation at 94 °C followed by 28 (for *BnActin7*) or 36 (for *BnaA08g20960D*) cycles with each cycle composed of 94 °C for 30s, 58 °C for 30s and 72 °C for 30s. PCR products were then analyzed by 2.0% agarose gel electrophoresis. All primers are listed in (Additional file 20: Table S6). Each experiment was biologically repeated three times.

## Supplementary Information

**Additional file 1: Figure S1.** Phenotype of one-week-old wild type (WT) and *bnd2* seedlings.

**Additional file 2: Figure S2.** Plants of WT and *bnd2* at 5-week-old seedling stage.

**Additional file 3: Figure S3.** Leaves of WT and *bnd2* at 5-week-old seedling stage.

**Additional file 4: Figure S4.** Plants of WT and *bnd2* at peak flowering stage.

**Additional file 5: Figure S5.** Whole plant phenotype of WT and *bnd2*.

**Additional file 6: Table S1.** Agronomic characters of WT and *bnd2*.

**Additional file 7: Figure S6.** Plant internodes of WT and *bnd2*.

**Additional file 8: Figure S7.** Phenotype of siliques of WT and *bnd2. (DOCX 94 kb)*

**Additional file 9: Figure S8.** Phenotypes of WT (left), *bnd2* (right) and their F_1_ hybrid (middle) at maturity.

**Additional file 10: Figure S9.** Plant internodes of WT (left), *bnd2* (right) and their F_1_ hybrid (middle) at maturity.

**Additional file 11: Figure S10.** Phenotype and trait inheritance of *bnd2* in the cross population.

**Additional file 12: Table S2.** Statistics of the sequencing datasets.

**Additional file 13: Figure S11.** The genotypes of some F_2:3_ lines derived from cross between *bnd2* and L329 at the marker ID1656.

**Additional file 14: Figure S12**. qRT-PCR analysis showing the mRNA expression levels of *BnaA08g20960D* in WT and *bnd2*.

**Additional file 15: Figure S13.** semi-qPCR analysis showing the mRNA expression pattern of *BnaA08g20960D* in WT and *bnd2*.

**Additional file 16: Figure S14.** Phenotypes of L329 (left), *bnd2* (right) and their hybrid (F_1,_ middle) at the maturation stage.

**Additional file 17: Table S3.** Agronomic characters of heterozygous F_1_ between L329 and *bnd2*.

**Additional file 18: Table S4.** Primer sequences for designed markers.

**Additional file 19: Table S5.** Primer sequences for qRT-PCR.

**Additional file 20: Table S6.** Primer sequences for semi-qPCR.

## Data Availability

All data generated or analyzed during this study are included in this published article (and its additional files). The original sequencing datasets of BSA-seq for *bnd2* have been deposited in the NCBI Sequence Read Archive (SRA, https://www.ncbi.nlm.nih.gov/sra) under accession numbers: SRR12968239, SRR12968240, SRR12968241, and SRR12968242. Any material generated during the current study is available from the corresponding author on reasonable request.

## Abbreviations

BND2: *Brassica napus dwarf* 2
EMS: ethyl methanesulfonate
BSA: bulked segregant analysis
BSA-seq: BSA and Next generation sequencing
SNP(s): single nucleotide polymorphism(s)
InDel(s): insertion(s) and deletion(s)
YPP: yield per plant
SPS: seeds per silique
SPP: siliques per plant
PH: plant height
IL: internode length
FBH: first branch height
MIL: main inflorescence length
NPB: number of effective primary branches
NSR: number of siliques on raceme
LS: length of siliques
TSW: thousand-seed weight
PCR: polymerase chain reaction
IPK1: Inositol 1,3,4,5,6-Pentapentaphosphate 2 kinase 1
