# Supplementary figures and images for: Characterization and fine mapping of a new dwarf mutant in Brassica napus
Source: BMC Plant Biol. 2021 Feb 26;21:117. doi: 10.1186/s12870-021-02885-y (PMC7908660; doi:10.1186/s12870-021-02885-y)

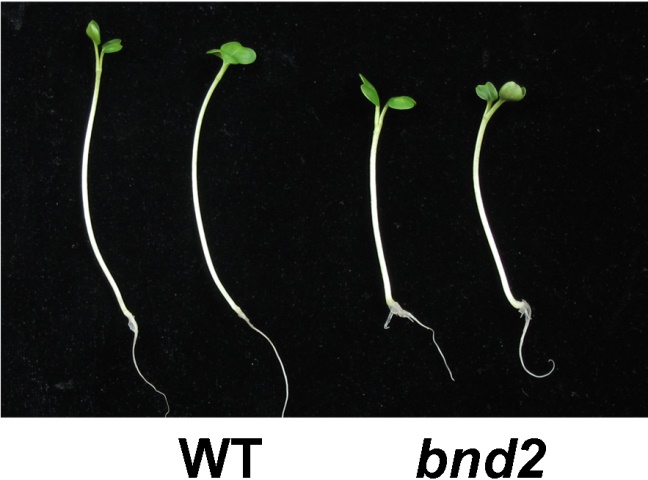


**Figure S1.** Phenotype of one-week-old wild type (WT) and *bnd2* seedlings.

Supplement: Supplementary file 1 — Additional file 1: Figure S1. Phenotype of one-week-old wild type (WT) and bnd2 seedlings. [file 12870_2021_2885_MOESM1_ESM.docx]

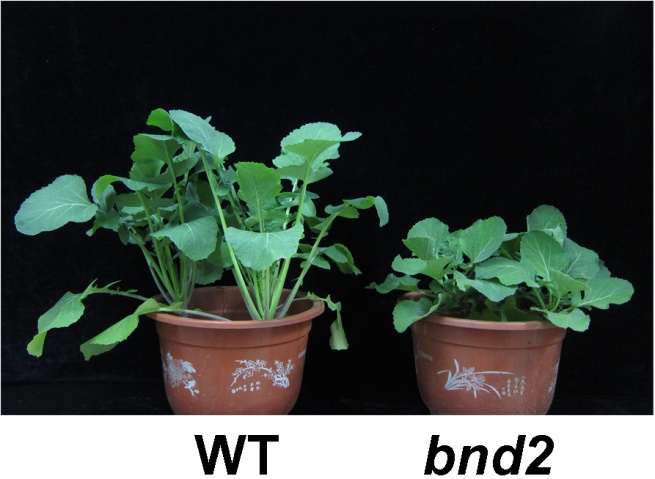


**Figure S2.** Plantsof WT and *bnd2* at 5-week-old seedling stage.

Supplement: Supplementary file 2 — Additional file 2: Figure S2. Plants of WT and bnd2 at 5-week-old seedling stage. [file 12870_2021_2885_MOESM2_ESM.docx]

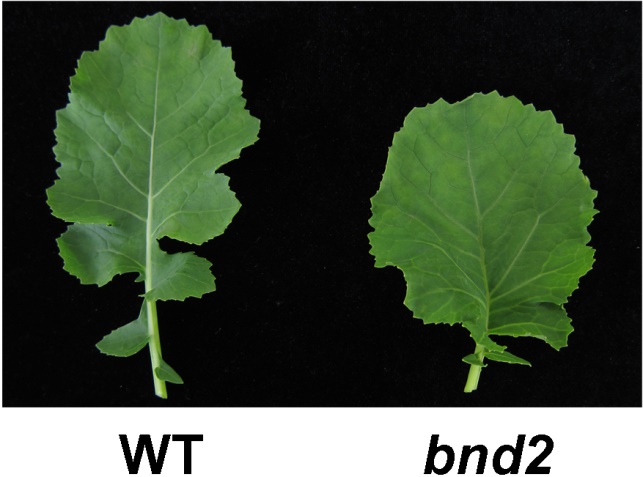


**Figure S3.** Leaves of WT and *bnd2* at 5-week-old seedling stage.

Supplement: Supplementary file 3 — Additional file 3: Figure S3. Leaves of WT and bnd2 at 5-week-old seedling stage. [file 12870_2021_2885_MOESM3_ESM.docx]

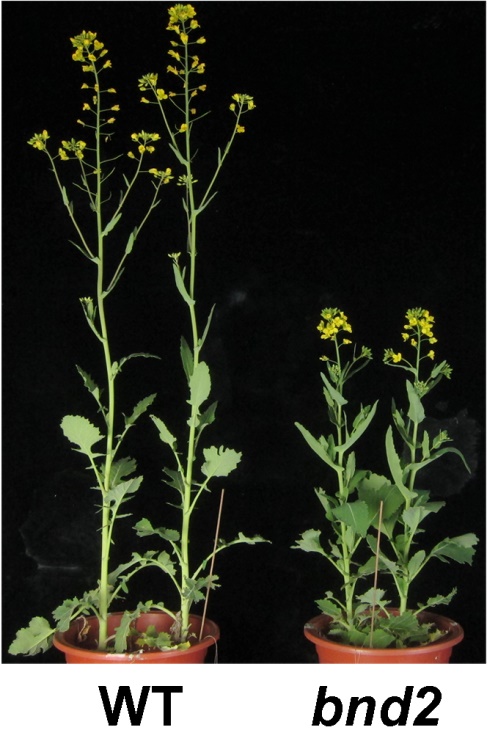


**Figure S4.** Plants of WT and *bnd2* at peak flowering stage.

Supplement: Supplementary file 4 — Additional file 4: Figure S4. Plants of WT and bnd2 at peak flowering stage. [file 12870_2021_2885_MOESM4_ESM.docx]

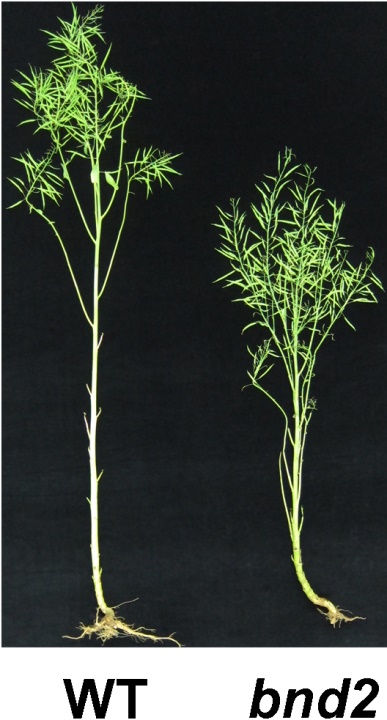


**Figure S5.** Whole plant phenotype ofWT and *bnd2*.

Supplement: Supplementary file 5 — Additional file 5: Figure S5. Whole plant phenotype of WT and bnd2. [file 12870_2021_2885_MOESM5_ESM.docx]

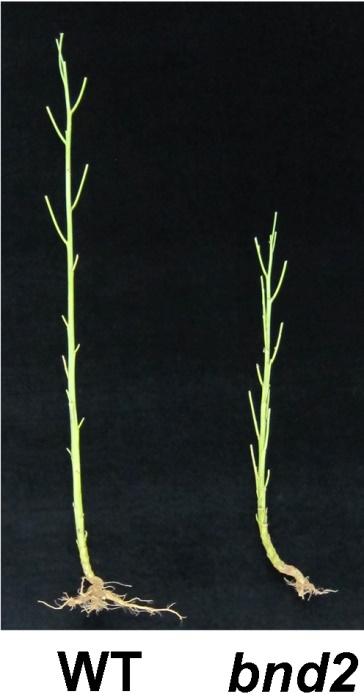


**Figure S6.** Plant internodes of WT and *bnd2*.

Supplement: Supplementary file 7 — Additional file 7: Figure S6. Plant internodes of WT and bnd2. [file 12870_2021_2885_MOESM7_ESM.docx]

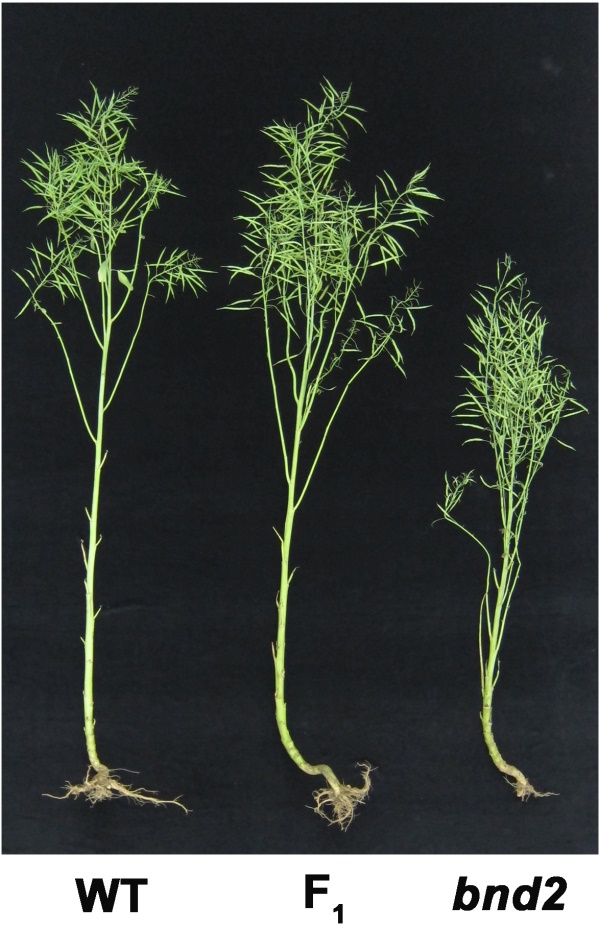


**Figure S8.** Phenotypes of WT (left), *bnd2* (right) and their F1 hybrid (middle) at maturity.

Supplement: Supplementary file 9 — Additional file 9: Figure S8. Phenotypes of WT (left), bnd2 (right) and their F1 hybrid (middle) at maturity. [file 12870_2021_2885_MOESM9_ESM.docx]

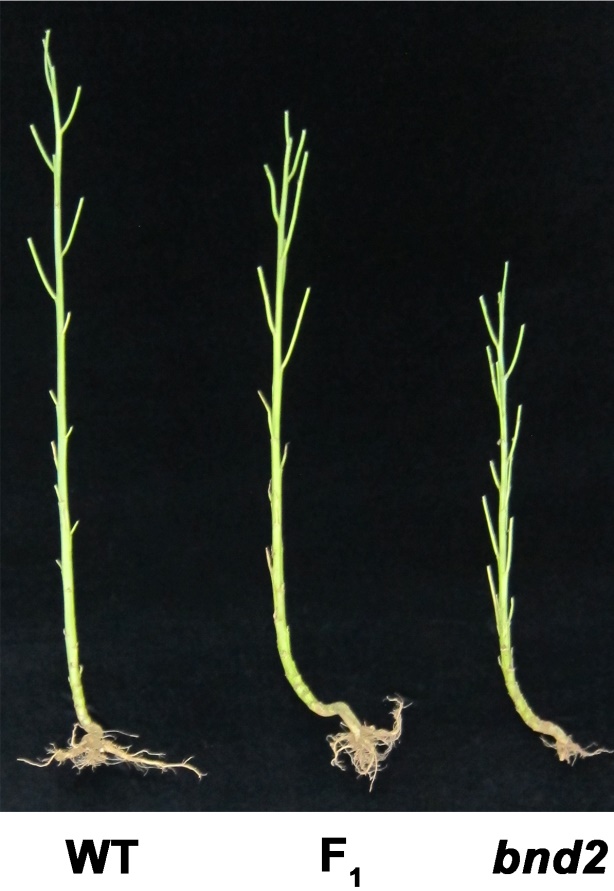


**Figure S9.** Plant internodes of WT (left), *bnd2* (right) and their F1 hybrid (middle) at maturity.

Supplement: Supplementary file 10 — Additional file 10: Figure S9. Plant internodes of WT (left), bnd2 (right) and their F1 hybrid (middle) at maturity. [file 12870_2021_2885_MOESM10_ESM.docx]

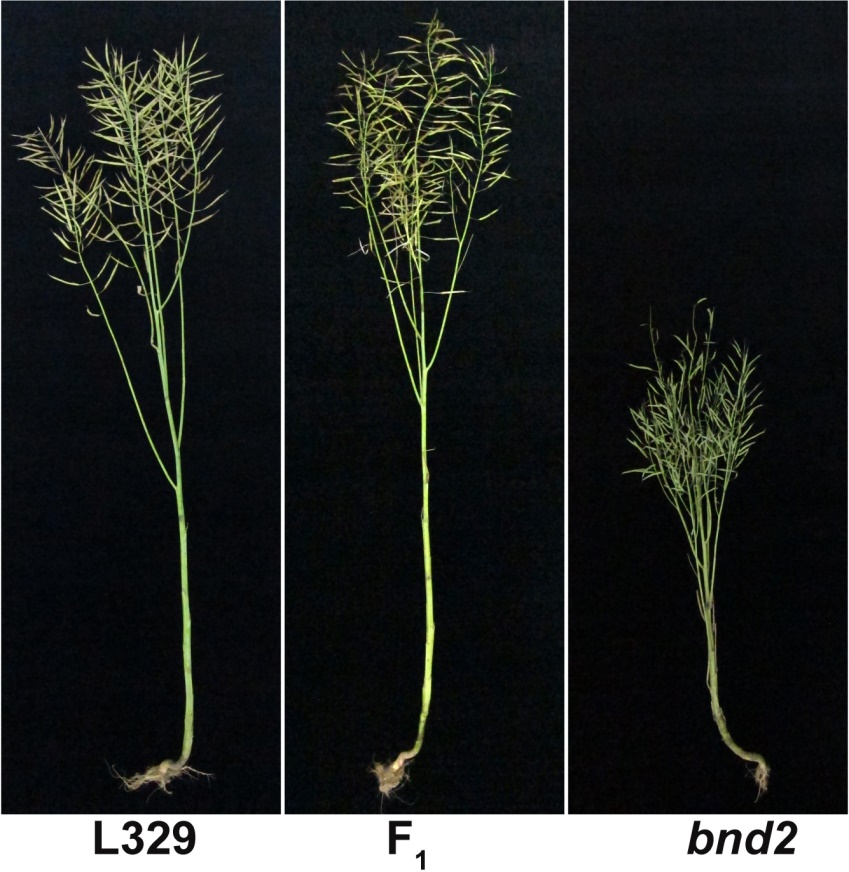


**Figure S14.** Phenotypes of L329 (left), *bnd2* (right) and their hybrid (F1, middle) at the maturation stage.

Supplement: Supplementary file 16 — Additional file 16: Figure S14. Phenotypes of L329 (left), bnd2 (right) and their hybrid (F1, middle) at the maturation stage. [file 12870_2021_2885_MOESM16_ESM.docx]
