# Supplementary material for: Characterization and fine mapping of a new dwarf mutant in Brassica napus
Source: BMC Plant Biol. 2021 Feb 26;21:117. doi: 10.1186/s12870-021-02885-y (PMC7908660; doi:10.1186/s12870-021-02885-y)
Supplement: Supplementary file 6 — Additional file 6: Table S1. Agronomic characters of WT and bnd2. [file 12870_2021_2885_MOESM6_ESM.docx]

**Table S1.** Agronomic characters of WT and *bnd2*

(‾*x*±*s*)

|  | PH/cm | FBH/cm | NPB | MIL/cm |
| --- | --- | --- | --- | --- |
| WT | 168.2±7.6 | 60.8±9.7 | 7.6±1.4 | 62.1±4.9 |
| *bnd2* | 100.7±8.1*** | 25.0±3.2*** | 5.8±1.1* | 52.9±7.7* |
|  | NSR | SPP | LS/cm | SPS |
| WT | 61.1±3.9 | 255.7±69.9 | 8.3±0.4 | 27.8±3.1 |
| *bnd2* | 49.5±10.3* | 169.8±57.6 | 6.9±0.4** | 25.6±3.6** |
|  | IL/cm | IN | TSW/g | YPP/g |
| WT | 8.6±0.7 | 19.7±1.9 | 3.4±0.1 | 18.8±6.2 |
| *bnd2* | 6.6±1.5*** | 13.6±2.1*** | 2.7±0.2** | 9.1±2.0** |

Notes: PH, plant height; FBH, first branch height; NPB, number of effective primary branches; MIL, main inflorescence length; NSR, number of siliques on raceme; SPP, siliques per plant; LS, length of siliques; SPS, seeds per silique; IL, internode length; IN, internode number; TSW, thousand-seed weight; YPP, yield per plant.‾*x*, Mean; *s*, Standard deviation; *n*=10; The significance of difference was determined by Student’s *t*-test (*, *P*<0.05; **, *P*<0.01; ***, *P*<0.001).
