# Supplementary material for: Characterization and fine mapping of a new dwarf mutant in Brassica napus
Source: BMC Plant Biol. 2021 Feb 26;21:117. doi: 10.1186/s12870-021-02885-y (PMC7908660; doi:10.1186/s12870-021-02885-y)
Supplement: Supplementary file 8 — Additional file 8: Figure S7. Phenotype of siliques of WT and bnd2. (DOCX 94 kb) [file 12870_2021_2885_MOESM8_ESM.docx]

**
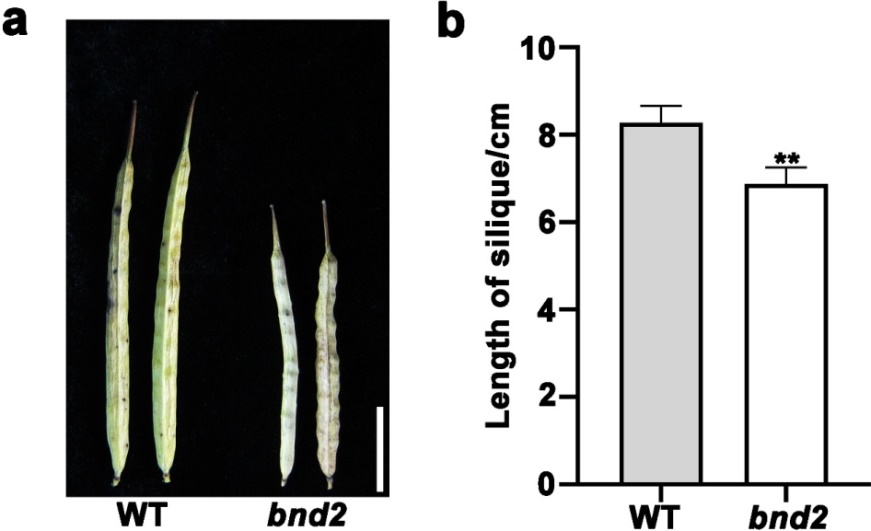
**

**Figure S7.** Phenotype of siliques of WT and *bnd2*. **a-b** Comparison of siliques of WT and *bnd2* at maturation stage. Value in **(b)** is shown as mean ± SD (*n*=10). Bar=2 cm. The significance of difference was determined by Student’s *t-*test (**, *P*<0.01).
