# Supplementary material for: Characterization and fine mapping of a new dwarf mutant in Brassica napus
Source: BMC Plant Biol. 2021 Feb 26;21:117. doi: 10.1186/s12870-021-02885-y (PMC7908660; doi:10.1186/s12870-021-02885-y)
Supplement: Supplementary file 11 — Additional file 11: Figure S10. Phenotype and trait inheritance of bnd2 in the cross population. [file 12870_2021_2885_MOESM11_ESM.docx]

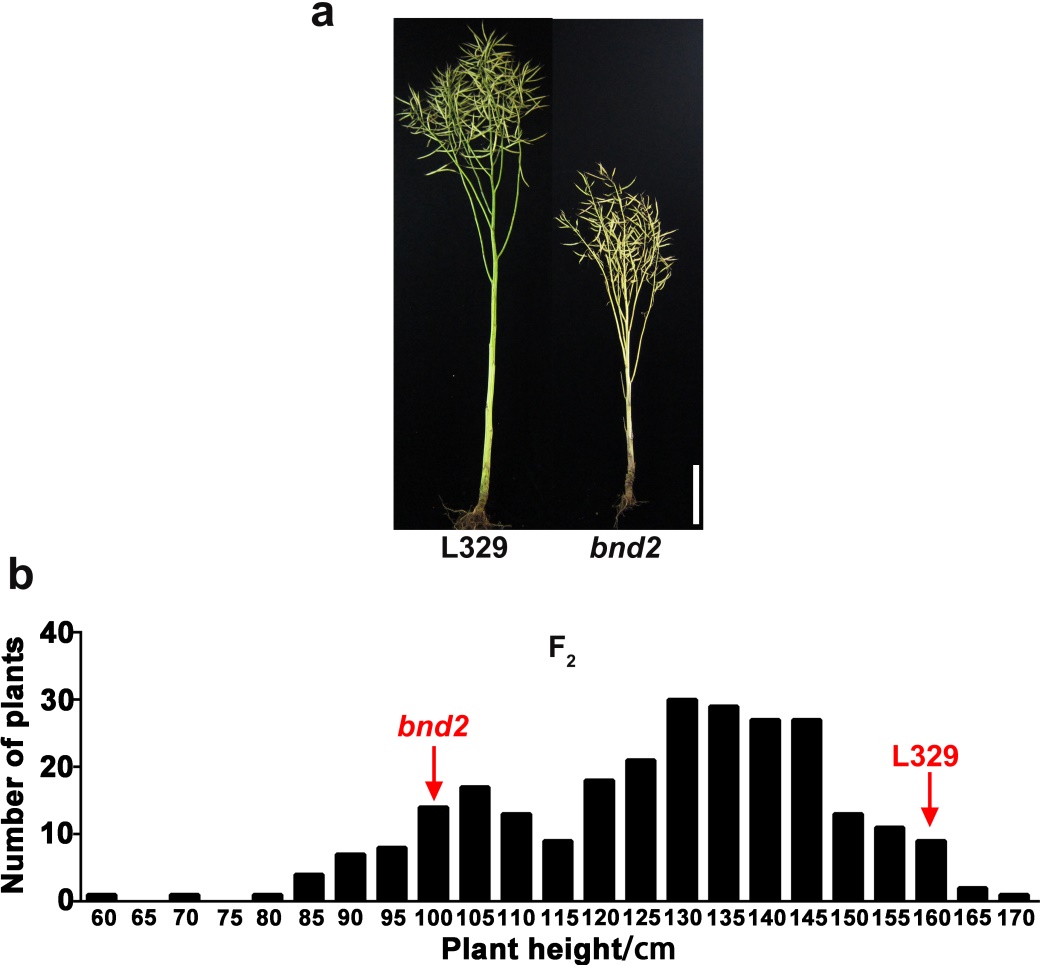


**Figure S10.** Phenotype and trait inheritance of *bnd2* in the cross population. **a** Phenotype of L329 and *bnd2* at maturity. Bar=20 cm. **b** Plant height frequency distribution of F_2_ population of 263 individuals derived from the cross of *bnd2* and L329.
