# Supplementary material for: Characterization and fine mapping of a new dwarf mutant in Brassica napus
Source: BMC Plant Biol. 2021 Feb 26;21:117. doi: 10.1186/s12870-021-02885-y (PMC7908660; doi:10.1186/s12870-021-02885-y)
Supplement: Supplementary file 12 — Additional file 12: Table S2. Statistics of the sequencing datasets. [file 12870_2021_2885_MOESM12_ESM.docx]

**Table S2.** Statistics of the sequencing datasets

| Sample | Clean bases | Clean reads | Q30 (%) | Q20 (%) | GC (%) | Genome coverage (%) | Mean depth (X) |
| --- | --- | --- | --- | --- | --- | --- | --- |
| L329 | 31 213 335 476 | 105 361 953 | 93.06 | 97.82 | 36.79 | 73.69 | 24.33 |
| *bnd2* | 26 109 039 838 | 89 416 611 | 95.43 | 98.49 | 36.76 | 72.69 | 18.55 |
| HB | 29 093 043 812 | 99 097 181 | 95.71 | 98.61 | 38.08 | 75.95 | 21.41 |
| SB | 32 337 768 132 | 109 214 266 | 92.99 | 97.78 | 37.78 | 75.96 | 22.35 |
| Mean | - | - | - | - | 37.75 | 74.57 | 21.66 |

Notes: clean bases: filtered bases number; clean reads: filtered reads number; Q30 (%): bases with mass values greater than or equal to 30 as a percentage of the total number of bases; Q20 (%): bases with mass values greater than or equal to 20 as a percentage of the total number of bases; GC (%): GC content of sample; Genome average (%): the number of bases covered by the sequence in the reference genome as a percentage of the genome; mean depth (X): average coverage depth.
