# Supplementary material for: Characterization and fine mapping of a new dwarf mutant in Brassica napus
Source: BMC Plant Biol. 2021 Feb 26;21:117. doi: 10.1186/s12870-021-02885-y (PMC7908660; doi:10.1186/s12870-021-02885-y)
Supplement: Supplementary file 13 — Additional file 13: Figure S11. The genotypes of some F2:3 lines derived from cross between bnd2 and L329 at the marker ID1656. [file 12870_2021_2885_MOESM13_ESM.docx]

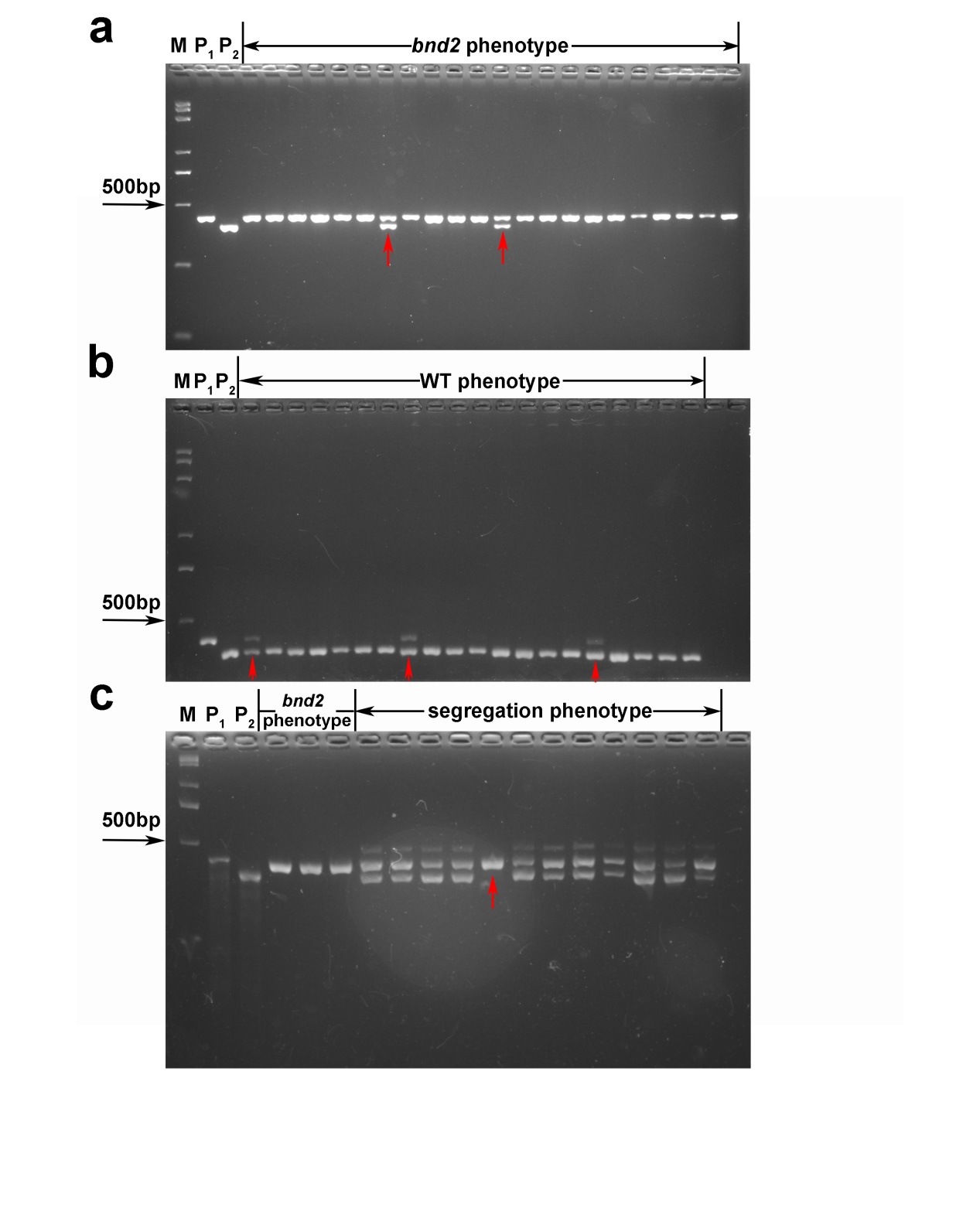


**Figure S11.** The genotypes of some F_2:3_ lines derived from cross between *bnd2* and L329 at the marker ID1656. **a** The genotypes of the lines with the *bnd2* phenotype. **b** The genotypes of the lines with WT phenotype. **c** The genotype of the lines with phenotype segregation. M means DNA Marker. P_1_ means the mutant parent *bnd2*. P_2_ means the WT parent L329. The red arrow indicates the recombinants between *BND2* and the marker ID1656.
