# Supplementary material for: Characterization and fine mapping of a new dwarf mutant in Brassica napus
Source: BMC Plant Biol. 2021 Feb 26;21:117. doi: 10.1186/s12870-021-02885-y (PMC7908660; doi:10.1186/s12870-021-02885-y)
Supplement: Supplementary file 14 — Additional file 14: Figure S12. qRT-PCR analysis showing the mRNA expression levels of BnaA08g20960D in WT and bnd2. [file 12870_2021_2885_MOESM14_ESM.docx]

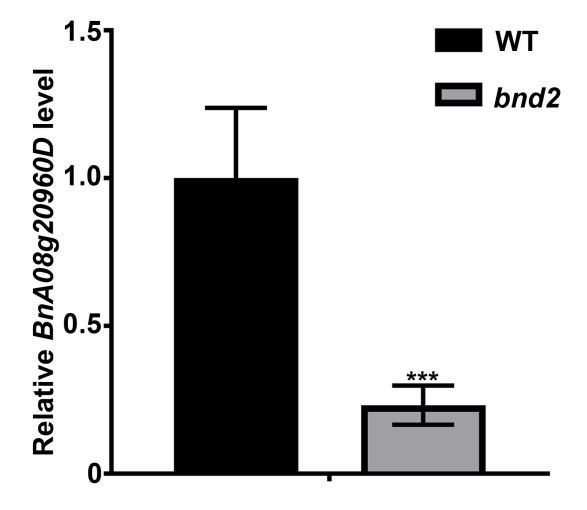


**Figure S12.** qRT-PCR analysis showing the mRNA expression levels of *BnaA08g20960D* in WT and *bnd2*. Seven-day-old WT and *bnd2* seedlings grown in soil were sampled for RNA analysis. *BnActin7* served as the internal control.Value is shown as mean ± SD (*n*=3). The significance of difference was determined by Student’s *t-*test (***, *P*<0.001).
