# Supplementary material for: Characterization and fine mapping of a new dwarf mutant in Brassica napus
Source: BMC Plant Biol. 2021 Feb 26;21:117. doi: 10.1186/s12870-021-02885-y (PMC7908660; doi:10.1186/s12870-021-02885-y)
Supplement: Supplementary file 15 — Additional file 15: Figure S13. semi-qPCR analysis showing the mRNA expression pattern of BnaA08g20960D in WT and bnd2. [file 12870_2021_2885_MOESM15_ESM.docx]

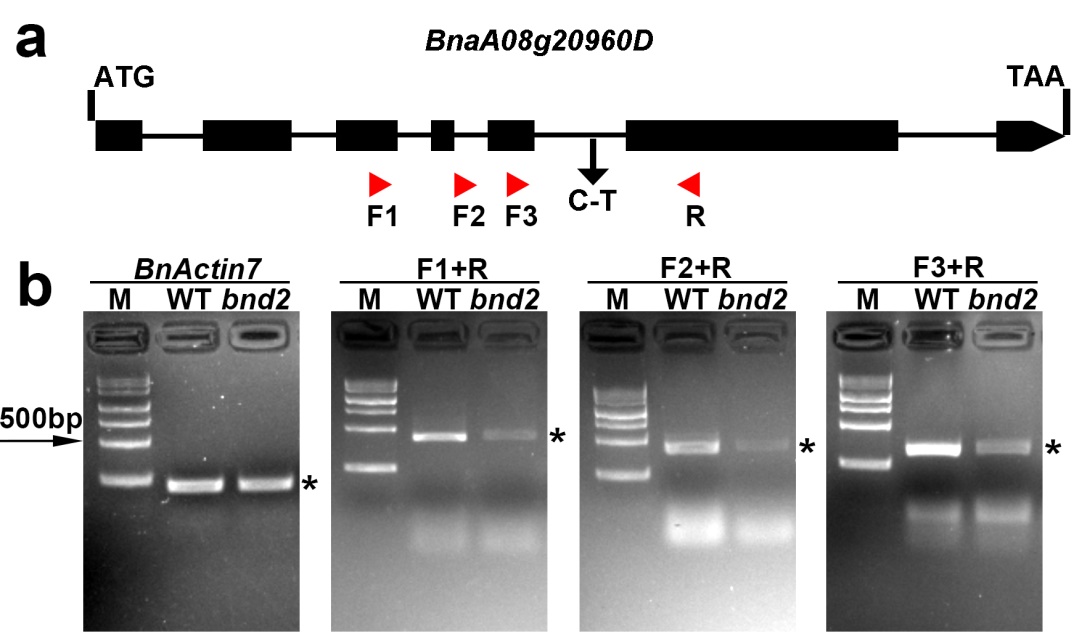


**Figure S13.** semi-qPCR analysis showing the mRNA expression pattern of *BnaA08g20960D* in WT and *bnd2*. **a** Structure of the *BnaA08g20960D* gene, a single nucleotide substitution (C-T) between *bnd2* and its wild type parent 2B in the fifth intron. Exons and introns were represented as black boxes or black lines, respectively. Red triangles show the position of primers used by semi-qPCR. **b** semi-qPCR analysis showing the mRNA expression pattern of *BnaA08g20960D* in WT and *bnd2*. Seven-day-old WT and *bnd2* seedlings grown in soil were sampled for RNA analysis. *BnActin7* was used as the internal control. M means DNA Marker. The asterisk represents the PCR product. The experiments were biologically repeated three times with similar results.
