# Supplementary material for: Characterization and fine mapping of a new dwarf mutant in Brassica napus
Source: BMC Plant Biol. 2021 Feb 26;21:117. doi: 10.1186/s12870-021-02885-y (PMC7908660; doi:10.1186/s12870-021-02885-y)
Supplement: Supplementary file 17 — Additional file 17: Table S3. Agronomic characters of heterozygous F1 between L329 and bnd2. [file 12870_2021_2885_MOESM17_ESM.docx]

**Table S3.** Agronomic characters of heterozygous F_1_ between L329 and *bnd2*

(‾*x*±*s*)

|  | PH/cm | FBH/cm | NPB | MIL/cm | NSR |
| --- | --- | --- | --- | --- | --- |
| L329 | 182.3±5.7^a^ | 72.5±8.7^a^ | 5.6±0.9^a^ | 73.9±8.3^a^ | 76.4±8.1^a^ |
| F_1_ | 181.8±7.4^a^ | 82.4±7.4^b^ | 5.4±0.8^a^ | 65.8±4.1^b^ | 75.4±6.0^a^ |
| *bnd2* | 114±3.7^b^ | 23.8±2.6^c^ | 5.8±0.8^a^ | 46.8±0.8^c^ | 46.3±1.3^b^ |
|  | SPP | LS/cm | SPS | TSW/g | YPP/g |
| L329 | 289.8±33.8^a^ | 7.4±0.4^a^ | 23.1±1.6^a^ | 4.7±0.8^a^ | 16.2±3.3^a^ |
| F_1_ | 254.4±58.7^a^ | 7.7±0.4^a^ | 25.4±2.0^b^ | 4.4±0.6^b^ | 21.5±1.8^b^ |
| *bnd2* | 160.8±20.5^b^ | 6.1±0.3^b^ | 22.1±1.9^a^ | 3.3±0.7^c^ | 7.1±1.9^c^ |

Notes: PH, plant height; FBH, first branch height; NPB, number of effective primary branches; MIL, main inflorescence length; NSR, number of siliques on raceme; SPP, siliques per plant; LS, length of siliques; SPS, seeds per silique; TSW, thousand-seed weight; YPP, yield per plant.‾*x*, Mean; *s*, Standard deviation; *n*=10; The significance of difference was determined by Student’s *t-*test and the significant differences were shown in a, b, c.
