# Supplementary material for: Characterization and fine mapping of a new dwarf mutant in Brassica napus
Source: BMC Plant Biol. 2021 Feb 26;21:117. doi: 10.1186/s12870-021-02885-y (PMC7908660; doi:10.1186/s12870-021-02885-y)
Supplement: Supplementary file 18 — Additional file 18: Table S4. Primer sequences for designed markers. [file 12870_2021_2885_MOESM18_ESM.docx]

**Table S4.** Primer sequences for designed markers

| Name | 5’ primer | 3’ primer |
| --- | --- | --- |
| ID1421 | TTCTCCGTACTGACCTTGCTT | TTGTGTGGCATTGGTTTATGTT |
| ID1470 | GTTTCTATTCGACCACCTTCG | ATCAATGAACCAGCCCATTTA |
| ID1482 | ACGAGCTAAACTCAAAGATGACG | AATCTTCATCCTATCAGGCAAAC |
| ID1530 | GTGTTTAACTCGGGAGGAGAGA | ATGATTAGGACTAATCGGTGGC |
| ID1576 | TGAAATAATACCCTTGAGACAACC | GTGTGTAATCATTCAGTCCGTCA |
| ID1656 | TCTTCTGAGTACGAGCCATCTC | CATAACACTTTCTACGGGTATTGC |
| ID1667 | GAAATGCCAAAGCGAAGC | ACCGCCAACTAGGATCAGTC |
| SNP1540 | CTGGATTGTTATTGATTTTGCA | ACACACACACACACAAACATATACA |
| SNP1552 | GCCACTACCATACTTCACTAAGTAATT | AATGACTCCAAGAGGCAACAATA |
| SNP1553 | TCAGTGAATATGCGATCAACAT | ATGGTGCCTTTCTTTAGTCTTT |
| SNP1557 | GTAGCGGTAGGGATGTGGAAG | GCGATATTCAGCCGTTTAGTTT |
| SNP1562 | TGCAAGCTATCCAATCGGTTTAT | GGAGAAAACAATTCGTCTTAAGTGG |
