# Supplementary material for: Characterization and fine mapping of a new dwarf mutant in Brassica napus
Source: BMC Plant Biol. 2021 Feb 26;21:117. doi: 10.1186/s12870-021-02885-y (PMC7908660; doi:10.1186/s12870-021-02885-y)
Supplement: Supplementary file 19 — Additional file 19: Table S5. Primer sequences for qRT-PCR. [file 12870_2021_2885_MOESM19_ESM.docx]

**Table S5.** Primer sequences for qRT-PCR

| Name | 5’ primer | 3’ primer |
| --- | --- | --- |
| BnActin7 | TGAAGATCAAGGTGGTCGCA | AGAAGGCAGAAACACTTAGAAG |
| BnaA08g20960D | TACACTGGATCCTCTCCCTCTTTC | GCAAGAAACAAGTTCCTTGCAT |
