# Supplementary material for: Characterization and fine mapping of a new dwarf mutant in Brassica napus
Source: BMC Plant Biol. 2021 Feb 26;21:117. doi: 10.1186/s12870-021-02885-y (PMC7908660; doi:10.1186/s12870-021-02885-y)
Supplement: Supplementary file 20 — Additional file 20: Table S6. Primer sequences for semi-qPCR. [file 12870_2021_2885_MOESM20_ESM.docx]

**Table S6.** Primer sequences for semi-qPCR

| Name | 5’ primer | 3’ primer |
| --- | --- | --- |
| BnActin7 | TGAAGATCAAGGTGGTCGCA | AGAAGGCAGAAACACTTAGAAG |
| BnaA08g20960D-F1 | tgtggatactcaccgcaatt | TGCTCAAAGTCTTGTTCAACCTTG |
| BnaA08g20960D-F2 | TCGTGTTGAGGATAAACCATG | TGCTCAAAGTCTTGTTCAACCTTG |
| BnaA08g20960D-F3 | TGTGGATTCCTTCCATCTTCAAGT | TGCTCAAAGTCTTGTTCAACCTTG |
